# Requirement of FAT and DCHS protocadherins during hypothalamic-pituitary development

**DOI:** 10.1172/jci.insight.134310

**Published:** 2020-12-03

**Authors:** Emily J. Lodge, Paraskevi Xekouki, Tatiane S. Silva, Cristiane Kochi, Carlos A. Longui, Fabio R. Faucz, Alice Santambrogio, James L. Mills, Nathan Pankratz, John Lane, Dominika Sosnowska, Tina Hodgson, Amanda L. Patist, Philippa Francis-West, Françoise Helmbacher, Constantine A. Stratakis, Cynthia L. Andoniadou

**Affiliations:** 1 Centre for Craniofacial & Regenerative Biology, King’s College London, Guy’s Campus, London, United Kingdom.; 2 Pediatric Endocrinology Unit, Irmandade da Santa Casa de Misericórdia de São Paulo, São Paulo, Brazil.; 3 Section on Endocrinology and Genetics, *Eunice Kennedy Shriver* National Institute of Child Health and Human Development, National Institutes of Health (NIH), Bethesda, Maryland, USA.; 4 Department of Medicine III, University Hospital Carl Gustav Carus, Technische Universität Dresden, Dresden, Germany.; 5 Epidemiology Branch, Division of Intramural Population Health Research, *Eunice Kennedy Shriver* National Institute of Child Health and Human Development, NIH, Bethesda, Maryland, USA.; 6 Department of Laboratory Medicine and Pathology, University of Minnesota Medical School, Minneapolis, Minnesota, USA.; 7 Aix Marseille Université, CNRS, IBDM - UMR7288, Marseille, France.

**Keywords:** Development, Endocrinology, Embryonic development, Genetic diseases, Neuroendocrine regulation

## Abstract

Pituitary developmental defects lead to partial or complete hormone deficiency and significant health problems. The majority of cases are sporadic and of unknown cause. We screened 28 patients with pituitary stalk interruption syndrome for mutations in the FAT/DCHS family of protocadherins that have high functional redundancy. We identified 7 variants, 4 of which are putatively damaging, in *FAT2* and *DCHS2* in 6 patients with pituitary developmental defects recruited through a cohort of patients with mostly ectopic posterior pituitary gland and/or pituitary stalk interruption. All patients had growth hormone deficiency, and 2 presented with multiple hormone deficiencies and small glands. FAT2 and DCHS2 were strongly expressed in the mesenchyme surrounding the normal developing human pituitary. We analyzed *Dchs2^–/–^* mouse mutants and identified anterior pituitary hypoplasia and partially penetrant infundibular defects. Overlapping infundibular abnormalities and distinct anterior pituitary morphogenesis defects were observed in *Fat4^–/–^* and *Dchs1^–/–^* mouse mutants, but all animal models displayed normal commitment to anterior pituitary cell types. Together our data implicate FAT/DCHS protocadherins in normal hypothalamic-pituitary development and identify *FAT2* and *DCHS2* as candidates underlying pituitary gland developmental defects such as ectopic pituitary gland and/or pituitary stalk interruption.

## Abstract 2

FAT and DCHS protocadherins are necessary for correct hypothalamic-pituitary development in mice and humans.

## Introduction

Developmental pituitary defects affect 0.5 in 100,000 live births and may lead to varying degrees of pituitary hormone deficiency (1, 2). Beyond biochemical confirmation of hormonal defects, diagnosis is based on magnetic resonance imaging (MRI) to identify a small or absent anterior pituitary, interrupted or absent pituitary stalk (pituitary stalk interruption syndrome, or PSIS), and ectopic posterior pituitary (EPP) (3).

During embryonic development, a region of the ventral diencephalon of the hypothalamus termed the infundibulum, and a region of the oral epithelium termed Rathke’s pouch (RP), evaginate toward each other to form the pituitary gland. These actions are mediated by an array of developmental signals as well as the action of the surrounding mesenchyme (for a review see ref. 4). RP gives rise to the anterior pituitary while the infundibulum gives rise to the posterior pituitary and the pituitary stalk, which connects the posterior lobe to the hypothalamus. The vast majority of cases with pituitary developmental defects are sporadic and of unknown cause; few cases appear to be familial, and they are often attributed to germline biallelic mutations in transcription factors involved in the development of the infundibulum (5–7). Recently a whole-exome sequencing (WES) screen of patients with such defects identified compound defects in *DCHS1* among other gene variants, indicating that protocadherins could be involved in pituitary development (8).

Cadherins represent a major family of adhesion molecules involved in tissue formation. As such they possess extracellular domains that facilitate binding, as well as an intracellular domain capable of associating with adapter and signaling proteins (9). FAT and DCHS protocadherins act as ligand-receptor pairs when expressed in adjacent cells and have been implicated both in planar cell polarity regulating cell movements such as convergence-extension and cell migration (10, 11), as well as regulation of YAP/TAZ independently of the Hippo kinase cascade (12–14), which controls tissue proliferation and stem cell activity (15).

In humans there are 4 *FAT* paralogues (*FAT1*, *FAT2*, *FAT3*, and *FAT4*) and 2 *DCHS* paralogues (*DCHS1* and *DCHS2*) (16). Mutations in *FAT4* are linked to Hennekam syndrome, while biallelic mutations in *FAT4* or *DCHS1* genes are associated with Van Maldergem syndrome (VMS). Besides symptoms common to both syndromes, such as intellectual disability and craniofacial malformations, VMS-specific clinical symptoms include camptodactyly, syndactyly, small kidneys, osteopenia, and tracheal abnormalities (14, 17–21), whereas lymphangiectasia and lymphedema are specific to Hennekam syndrome (17, 18, 22). A link between VMS and endocrine abnormalities, including hypogonadotropic hypogonadism and amazia, were reported in a recent study, providing new support to the possible involvement of FAT/DCHS signaling in hypothalamic-pituitary axis development or function (23). Null deletions of *Fat4* or *Dchs1* in mouse lead to overlapping phenotypes, including inner ear, neural tube, kidney, skeleton, lung, and heart defects (24), further supporting that these protocadherins act as a ligand-receptor pair. However, both mutant mice die shortly after birth, inhibiting the study of postnatal homozygous null animals (24, 25). *Dchs2^–/–^* mutant mice are viable and fertile, with analyses revealing functional redundancy between DCHS2 and DCHS1 (26). DCHS1 exhibits a broader expression pattern during development, and its loss generally results in more severe developmental phenotypes (16). We have previously characterized expression patterns of *Dchs1*, *Fat3*, and *Fat4* during murine embryonic development and identified expression in the hypothalamus, infundibulum, developing RP, and surrounding mesenchyme (27).

In this study we performed WES on 28 patients with EPP and/or PSIS and identified variants in *FAT2* and *DCHS2* predicted to be deleterious. We characterized expression of FAT2 and DCHS2 in the human developing gland and analyzed pituitary development in a series of FAT/DCHS mouse mutants (*Dchs1*, *Dchs2*, *Fat4*), which identified a range of infundibular and RP defects. Our data suggest a requirement for FAT and DCHS protocadherins in the infundibulum and mesenchyme surrounding the developing gland during morphogenesis, revealing that FAT/DCHS function is necessary for normal pituitary morphogenesis and their dysfunction can underlie developmental anomalies of the hypothalamic-pituitary axis.

## Results

### Molecular and in silico findings in patients with pituitary developmental defects.

**Figure 1:**
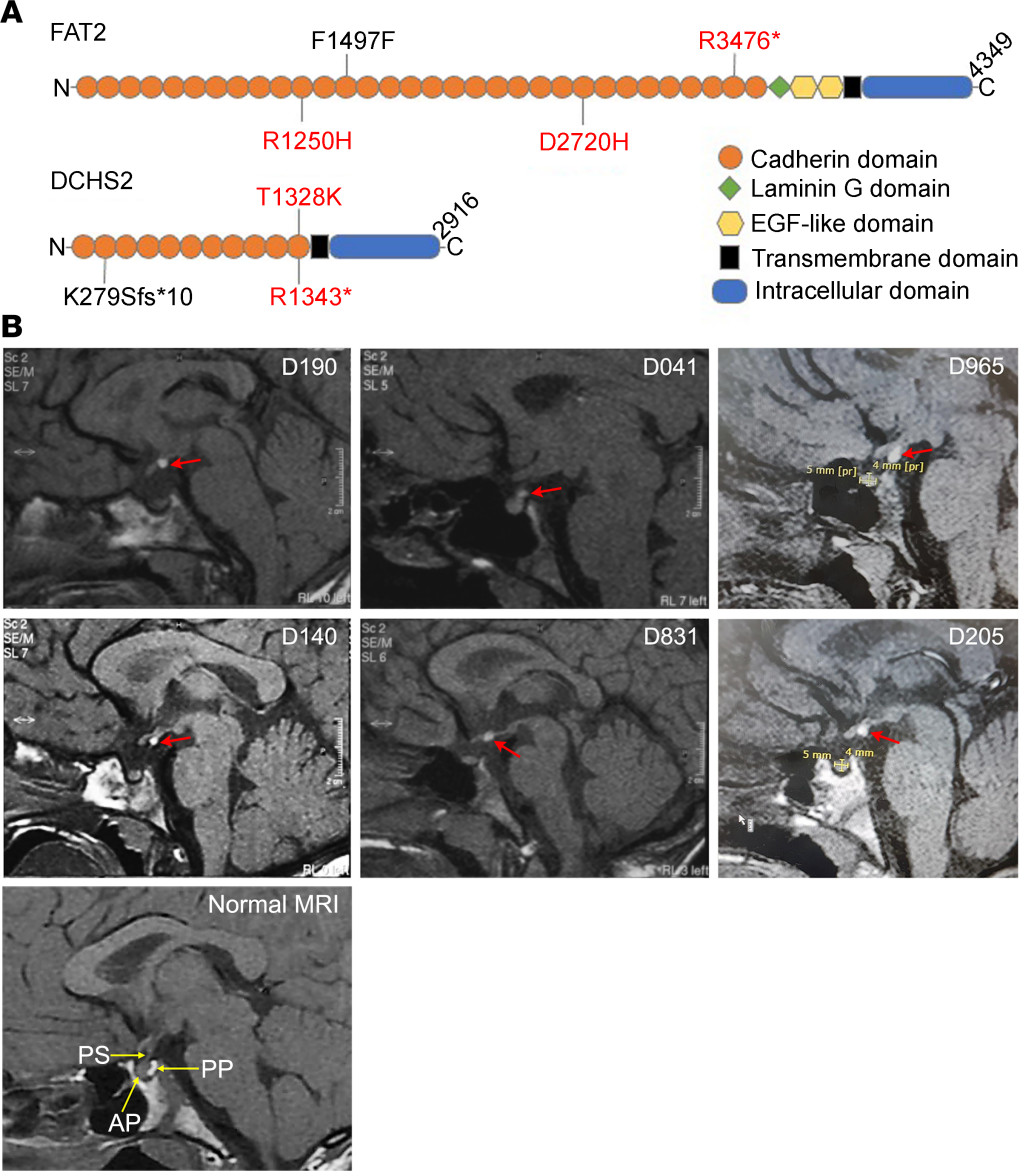
Variants in FAT2 and DCHS2 in patients with PSIS. (**A**) Representative schematic of the FAT2 and DCHS2 proteins indicating the locations of identified mutations. Variants in red are predicted as likely deleterious by Combined Annotation Dependent Depletion (CADD) score (>20). No CADD score data available for DCHS2 p.K279Sfs*10. (**B**) Sagittal T1 pituitary MRIs of the 6 patients with *FAT2*/*DCHS2* variants and normal MRI for comparison, bottom left. For each patient, the normal posterior pituitary bright spot is not seen in the pituitary fossa, but rather, an ectopic small region of high T1 signal at the top of the infundibulum or higher (red arrows). PS, pituitary stalk; AP, anterior pituitary; PP, posterior pituitary (normal intrasellar).

Combined with our description of expression of FAT/DCHS family members during pituitary development (27), the recent identification of links between DCHS abnormalities and pituitary developmental defects (8, 23) suggested an involvement of FAT/DCHS signaling in hypothalamic and/or pituitary development. To address this possibility, we studied 28 patients with congenital pituitary abnormalities of EPP and/or PSIS with WES, focusing on all 4 *FAT* genes, as well as *DCHS1* and *DCHS2*. Three heterozygous variants in DCHS2 and 4 in FAT2 were identified and their locations on the proteins summarized in a schematic (Figure 1A). The variants; their functional type, class (synonymous, nonsynonymous, or frameshift), effect (missense, nonsense, silent), and frequency in control database; and the genes in which these variants were identified in patients with EPP are presented in Table 1. The allele frequency of all variants in the cohort was 1.79%. Among all variants across these genes, only 2 variants in *DCHS2* and 1 variant in *FAT2* were classified as functionally “high” and predicted to be deleterious using prediction tools (see Methods) (Table 1).

### Clinical data.

The clinical and biochemical data of the 6 patients identified with *FAT2* and *DCHS2* variants are summarized in Table 2. Patient D190 was a 20.6-year-old male at the time of assessment, who was born full term with cryptorchidism and micropenis. He was diagnosed with multiple anterior pituitary hormone deficiencies and learning difficulties and received full hormone replacement therapy. MRI showed EPP and a very small anterior pituitary for his age and sex (Figure 1B). The patient required testosterone treatment for micropenis and for pubertal induction and maintenance. Despite long-term follow-up, he never presented any increment in testicular volume. He was found to harbor a *DCHS2* stop codon c.C4027T (p.R1343*) variant (Table 1). This was also found in the Atherosclerosis Risk in Communities Study (ARIC) controls and is reported as a rare variant in Genome Aggregation Database (gnomAD) and likely deleterious based on the CADD prediction tool (Table 1).

Patient D041 was a 16.4-year-old male who was born full term. He developed GHD and was treated with recombinant growth hormone (rGH) until the age of 16 years. His pituitary MRI revealed EPP and an anterior pituitary within the normal size range for his age and sex (Figure 1B). Molecular analysis revealed a synonymous *FAT2* variation F1497= (Table 1). This is reported as a very rare variant in gnomAD, although it is predicted to be likely benign based on CADD score (Table 1).

Patient D965 was a 13.9-year-old male at the time of assessment who was born at 34 weeks of gestation. He was diagnosed with GHD and MRI revealed an EPP (Table 2 and Figure 1B). He was still under rGH treatment during clinical assessment. Molecular analysis revealed a *FAT2* c.C10426T variant, which creates a stop codon at p.R3476*. This variant was not found in any of the controls, it is reported as a very rare variant in gnomAD, and it is likely deleterious according to the CADD prediction tool (Table 1). In addition, this patient was also found to harbor a variant in *DCHS2*: c.836_837delAAinsG (p.K279Sfs*10), which creates a frameshift resulting in premature termination of translation. This variant was not found in any of the controls and was absent from gnomAD (Table 1).

Patient D140 was a 20.7-year-old female who was born full term. She was diagnosed with GHD following hypoglycemic seizures and received treatment with rGH (Table 2). Pituitary MRI revealed EPP and possible PSIS (Figure 1B, Table 2). She was found to harbor a *DCHS2* missense variant (p.T1328K) predicted to be damaging by all 3 prediction tools that has not been reported previously to our knowledge (Table 1).

Patient D831 was a 15.1-year-old male who was born preterm at 31 weeks of gestation due to premature rupture of membranes. He was diagnosed with GH, thyrotropin hormone (TSH), and partial adrenocorticotropin hormone (ACTH) deficiency as well as learning difficulties. He received rGH therapy until the age of 15 years and is now on replacement with levothyroxine and steroids (Table 2). Pituitary MRI revealed EPP and possible PSIS (Figure 1B, Table 2). Molecular analysis revealed a *FAT2* p.R1250H missense variant absent from public databases and predicted as probably damaging by PolyPhen, damaging by Sorting Intolerant From Tolerant (SIFT), and likely deleterious by CADD. This was reported as very rare in gnomAD (Table 1).

Patient D205 was a 14.6-year-old male at the time of assessment. He was born at term with cryptorchidism and micropenis. He was diagnosed with GH and TSH deficiency and placed on replacement with rGH and levothyroxine (Table 2). He also required testosterone treatment for micropenis and for pubertal induction and maintenance. MRI revealed EPP (Figure 1). He was found to harbor a *FAT2* missense variant (p.D2720H), not reported previously to our knowledge, and predicted to be probably damaging by PolyPhen, damaging by SIFT, and likely deleterious by CADD (Table 1).

### DCHS2 and FAT2 are expressed during embryonic pituitary development.

**Figure 2:**
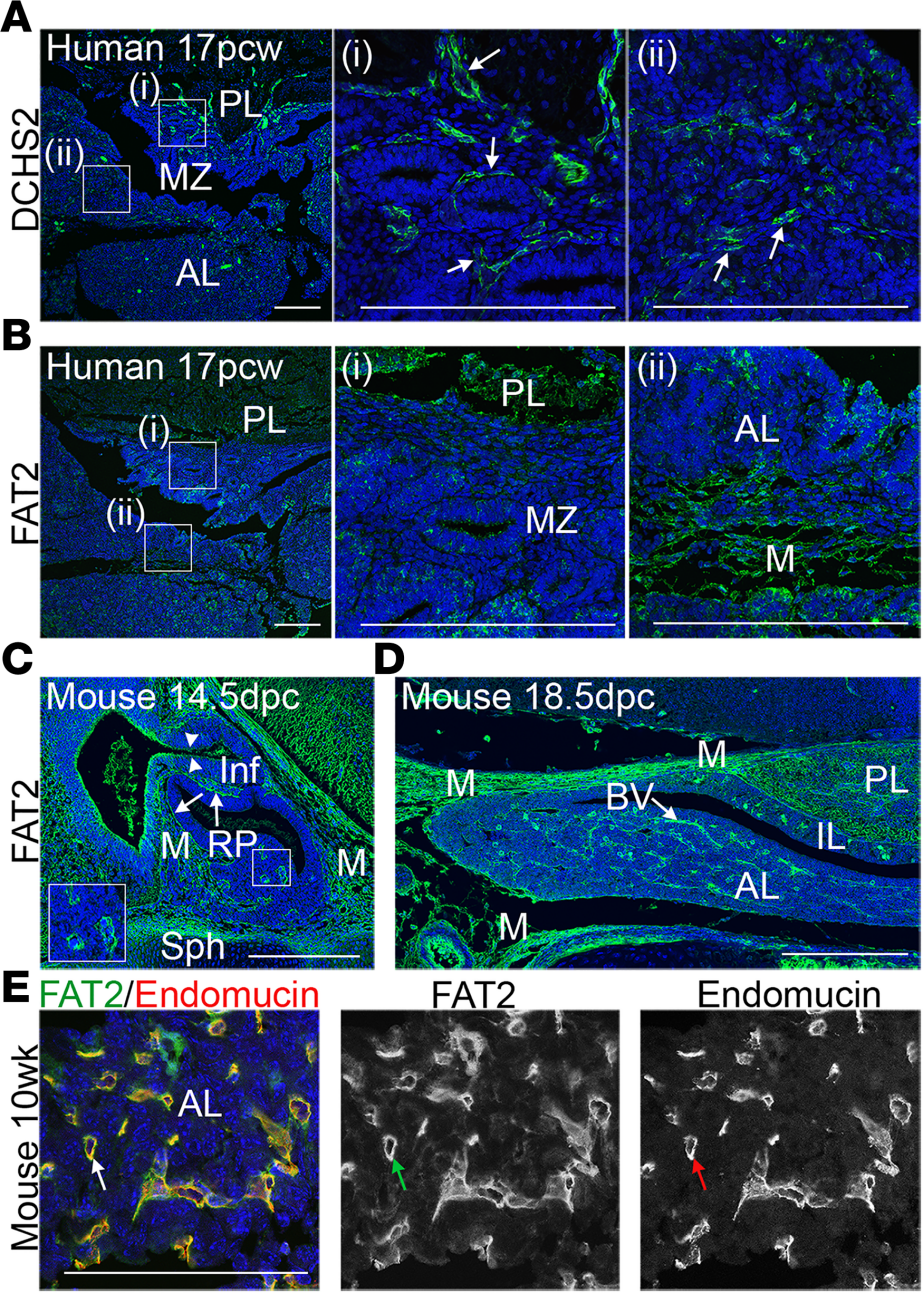
DCHS2 and FAT2 expression in the developing pituitary. (**A** and **B**) Immunofluorescence staining on 17 postconception week (pcw) human fetal pituitary sections, using specific antibodies against DCHS2 (**A**) and FAT2 (**B**); *n* = 2. DCHS2 is expressed in mesenchymal tissues surrounding epithelial structures in the marginal zone, between the posterior and anterior pituitary and within the anterior lobe (arrows). FAT2 localizes in mesenchymal tissues within the anterior lobe and is expressed throughout the posterior lobe, and diffuse staining is observed in epithelial structures in the marginal zone. Images in middle and at right are magnified boxed regions in **A** and **B**. (**C** and **D**) Immunofluorescence staining using antibodies against FAT2 on the developing mouse pituitary at 14.5 dpc (**C**) and 18.5 dpc (**D**); *n* = 3. At 14.5 dpc FAT2 localizes in mesenchymal tissue, in cells of RP making contact with the mesenchyme (arrows), and in cells of the infundibulum (arrowheads) (**C**). At 18.5 dpc, FAT2 expression persists in the mesenchyme surrounding the gland, throughout the posterior lobe, and in vasculature throughout the anterior lobe (**D**). (**E**) In the WT adult pituitary at 10 weeks, FAT2 (shown in green) is expressed strongly by cells of the vasculature, as seen by double immunofluorescence staining with Endomucin (shown in red) marking endothelial cells; *n* = 3. Scale bars: 200 μm (**A**–**C**), 100 μm (**E**). PL, posterior lobe; AL, anterior lobe; IL, intermediate lobe, MZ, marginal zone; Inf, infundibulum; M, mesenchyme; Sph, sphenoid bone; BV, blood vessels.

To explore whether the 2 genes contribute to pituitary development, we next examined expression of FAT2 and DCHS2 proteins in the developing human pituitary by immunostaining. At 17 postconception weeks (pcw), DCHS2 expression was detected in mesenchymal cells around epithelial structures of the marginal zone as well as in diffuse mesenchymal cells within the anterior lobe (arrows, Figure 2A). FAT2 expression was also detected in mesenchymal cells but also in the posterior lobe and at low levels in epithelial structures within the marginal zone (Figure 2B).

Specific cross-reactivity of the FAT2 antibody allowed us to carry out analysis in the mouse pituitary. At 14.5 dpc, FAT2 was abundantly detected in the mesenchyme surrounding the developing gland, in invading mesenchymal tissue that would form the vasculature (4, 28), as well as in cells at the external layer of the rostral RP (arrows) contacting the mesenchyme and in the infundibulum (arrowheads) (Figure 2C). Expression of *Fat2* and *Dchs2* in the mesenchyme surrounding the developing RP and infundibulum was confirmed by mRNA in situ hybridization (Supplemental Figure 1; supplemental material available online with this article; https://doi.org/10.1172/jci.insight.134310DS1). At 18.5 dpc just prior to birth, FAT2 expression remained strong in mesenchyme surrounding the pituitary; there was expression in the posterior lobe and abundant signal detected throughout the vasculature of the anterior lobe (Figure 2D). At 10 weeks in the adult mouse pituitary, FAT2 expression persisted in the vasculature as confirmed by double immunofluorescence staining with Endomucin, marking endothelial cells of the blood vessels (arrows, Figure 2E).

### Dchs2^–/–^ mouse mutants have defects in hypothalamic-pituitary development.

**Figure 3:**
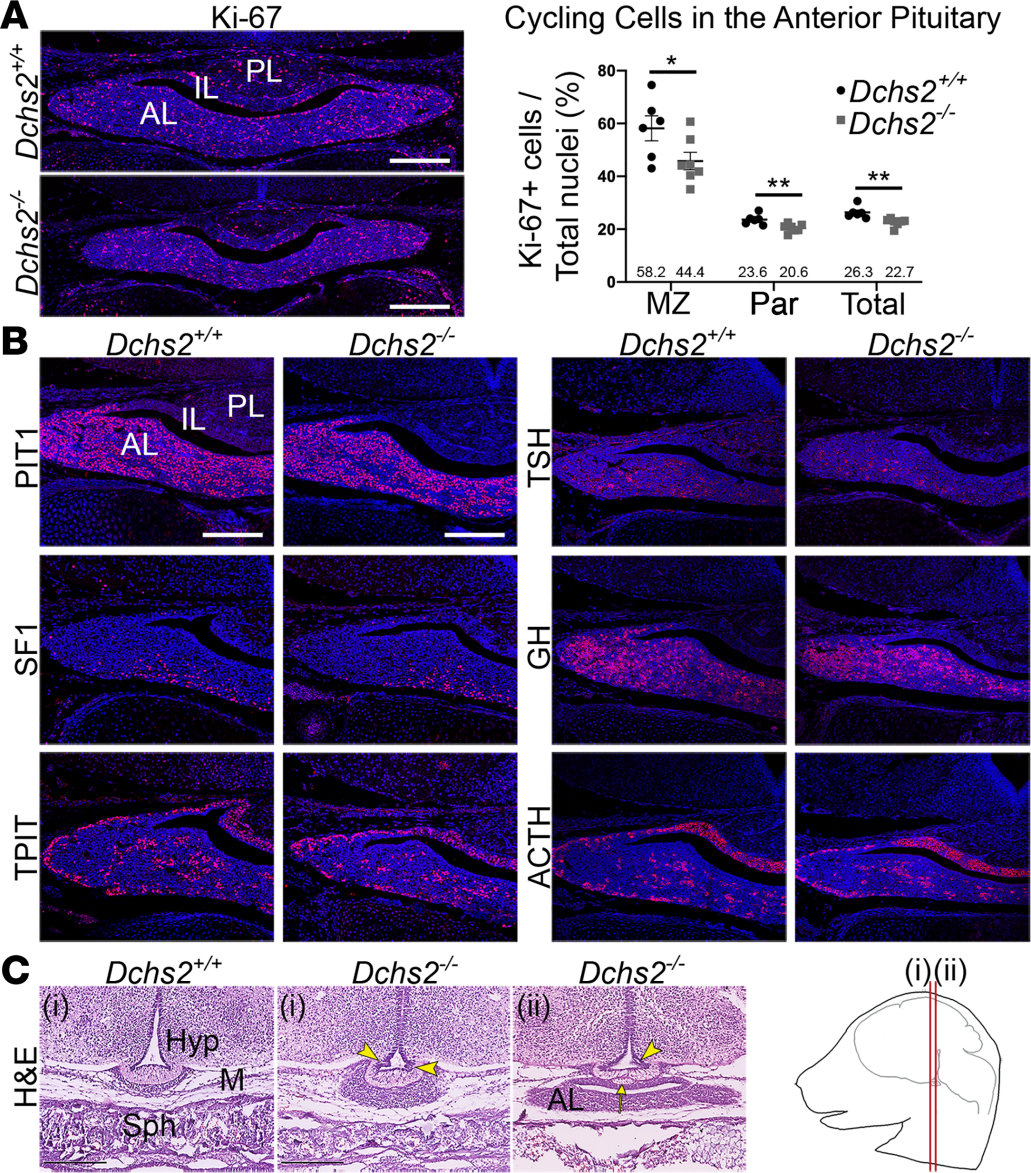
DCHS2 is required for normal murine pituitary development. (**A**) Immunofluorescence staining using antibodies against Ki-67 to detect cycling cells, on frontal sections of pituitaries from *Dchs2^–/–^* mutants and WT littermate controls at 18.5 dpc. Graph depicting quantification of cycling cells of the anterior pituitary of WT controls and *Dchs2^–/–^* mutants, showing reduced proliferation in mutants in the marginal zone (MZ) surrounding the cleft *P* = 0.0495, parenchyme (Par) *P* = 0.007, and total throughout the anterior lobe *P* = 0.0044, unpaired Student’s *t* test (*n* = 3 per genotype, multiple sections counted). Values of Ki-67–positive cells are expressed as a percentage of the total nuclei in the anterior lobe. Average values are indicated. *P < 0.05; **P < 0.01. (**B**) Immunofluorescence staining on *Dchs2^–/–^* pituitaries and littermate controls at 18.5 dpc (*n* = 3) using antibodies against lineage-committed progenitor markers PIT1 (thyrotrophs, somatotrophs, lactotrophs), SF1 (gonadotrophs), and TPIT (corticotrophs, melanotrophs) and hormones TSH, GH, and ACTH expressed in differentiated thyrotrophs, somatotrophs, and corticotrophs, respectively. Staining is comparable for all markers between genotypes. (**C**) Hematoxylin and eosin staining on frontal sections of *Dchs2^–/–^* mutants and controls at 18.5 dpc at axial levels as indicated in the cartoon (*n* = 6). Abnormal invaginations are seen in the median eminence (arrowheads) as well as lobulated protrusions (arrow). PL, posterior lobe; IL, intermediate lobe; AL, anterior lobe; Hyp, hypothalamus; M, mesenchyme; Sph, sphenoid bone. Scale bars: 200 μm (**A** and **B**), 250 μm (**C**).

Considering the functional link suggested by the identification of PSIS patients with putatively pathogenic *DCHS2* variants, we next sought to confirm such an implication using a *Dchs2* mutant mouse model. In order to confirm if DCHS2 has a function during hypothalamic-pituitary development, we analyzed *Dchs2^–/–^* null mutants. Gross inspection of the dissected mutant pituitary of neonates at P2 did not reveal any apparent morphological anomalies compared with controls (Supplemental Figure 2A, n** = 9). At 18.5 dpc, a stage when morphogenesis of the hypothalamic-pituitary axis has been fully achieved, and when PSIS-like anatomical phenotypes can be unambiguously detected, embryos exhibited mild anterior pituitary hypoplasia (3/6), accompanied by a significant reduction in the number of cycling cells as determined by antibody staining against Ki-67 (average 26.3% cycling cells in controls compared with 22.7% in mutants, *P* = 0.0044, *n* = 3, unpaired Student’s *t* test; Figure 3A). Analysis of cycling cells in the marginal zone surrounding the cleft and separately in the parenchyme revealed a reduction in both regions of mutants compared with controls (marginal zone *P* = 0.0495, parenchyme *P* = 0.007). We next sought to determine if anterior cell type commitment and differentiation occurred normally. Immunofluorescence staining using antibodies against commitment markers PIT1 (POU1F1), SF1 (NR5A1), and TPIT (TBX19) in *Dchs2^–/–^* and control *Dchs2^+/+^* pituitaries did not reveal differences between genotypes (Figure 3B), confirmed by quantitative reverse transcription PCR (qRT-PCR) (Supplemental Figure 2B, n** = 4). Similarly, no differences were observed for the expression of differentiation markers ACTH, GH, and TSH (*n* = 3, Figure 3B) or for the expression of the stem cell marker *Sox2* (Supplemental Figure 2B). These results are consistent with a role for DCHS2 in controlling morphogenesis rather than cell fate specification within the anterior pituitary.

Histological examination of frontal sections at 18.5 dpc at anterior axial levels revealed dysmorphology of the median eminence and developing pituitary stalk in a proportion of the mutants (4/6). Of note, the pituitary stalk in mice is very short compared with the developing human pituitary. Mutant embryos exhibited invaginations in the median eminence and the epithelium of the infundibular recess (arrowheads, Figure 3C, 2 mutant embryos shown, axial levels as indicated in the cartoon). None of these anomalies were observed in control littermates (*n* = 8).

### Fat4^–/–^ and Dchs1^–/–^ mutants exhibit additional morphogenetic defects of the anterior pituitary.

**Figure 4:**
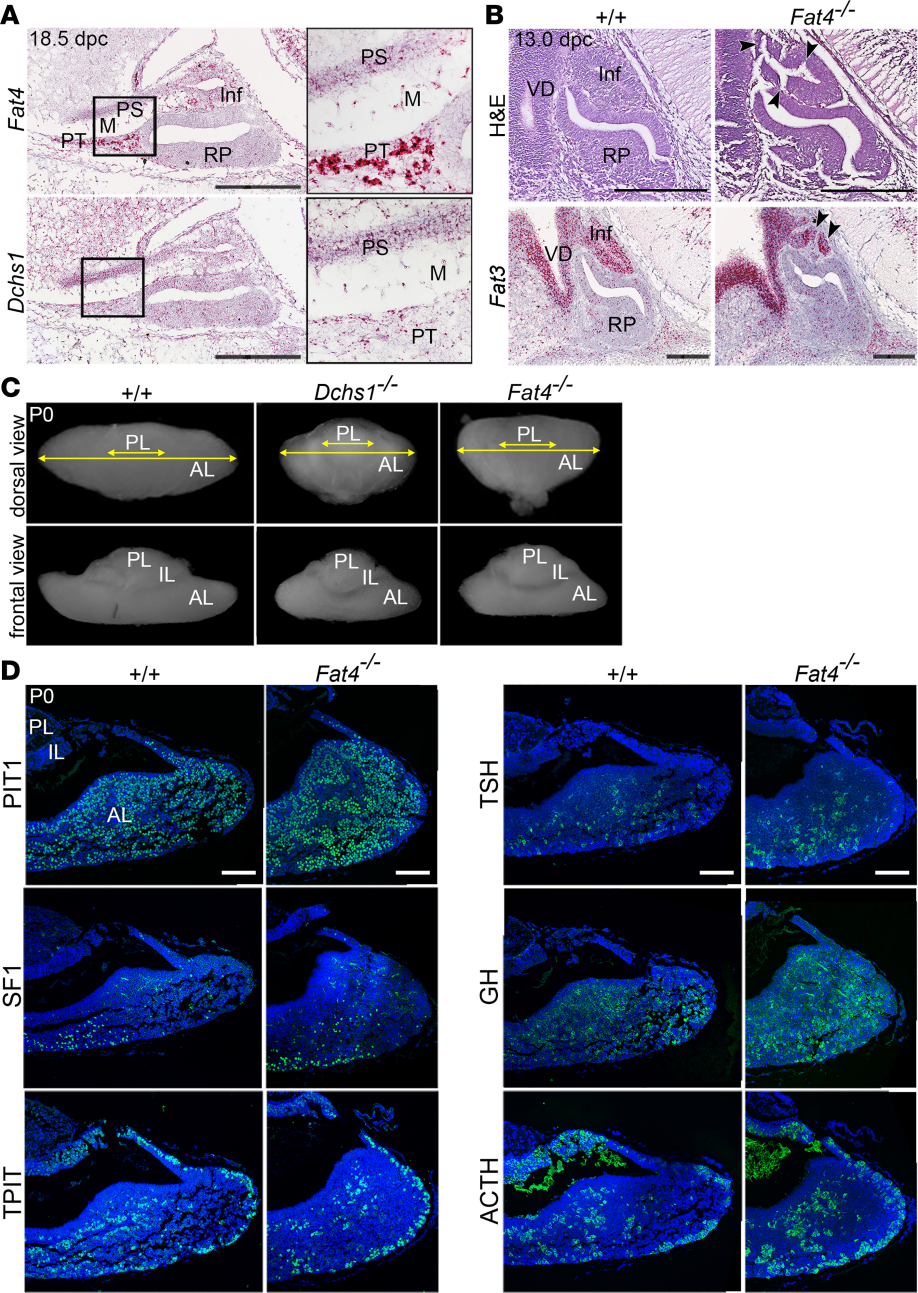
FAT4 and DCHS1 are required for normal murine pituitary development. (**A**) RNAscope mRNA in situ hybridization on sagittal sections through WT murine pituitaries at 18.5 dpc using probes against *Fat4* and *Dchs1* (*n* = 3). Abundant *Fat4* transcripts are detected in the pars tuberalis, infundibulum, developing pituitary stalk, and mesenchyme surrounding definitive RP. Some transcripts are also detected in RP. Expression of *Dchs1* is detected at low levels throughout these tissues. (**B**) Hematoxylin and eosin staining of sagittal sections through *Dchs1^–/–^* (*n* = 5), *Fat4^–/–^* (*n* = 10), and control pituitaries (*n* = 15) at 13.0 dpc showing invaginations in the infundibulum of *Fat4^–/–^* mutants (arrowheads), not observed in control or *Dchs1^–/–^* embryos. RNAscope mRNA in situ hybridization on sagittal sections through control WT and *Fat4^–/–^* pituitaries at 13.5 dpc using specific probes against *Fat3* marking the ventral diencephalon and infundibulum, which is abnormal in mutants (arrowheads). (**C**) Wholemount images taken at dorsal (top panels) and frontal views (bottom panels) of control, *Dchs1^–/–^* (*n* = 10), and *Fat4^–/–^* (*n* = 8) pituitaries at P0. Both *Dchs1^–/–^* and *Fat4^–/–^* mutants have a shortened medio-lateral axis affecting the anterior lobe compared with control. (**D**) Immunofluorescence staining on *Fat4^–/–^* pituitaries and littermate controls at 18.5 dpc using antibodies against lineage-committed progenitor markers PIT1, TPIT, and SF1 and hormones TSH, GH, and ACTH (*n* = 3). Staining is comparable for all markers between genotypes. Inf, infundibulum; PS, pituitary stalk; PT, pars tuberalis; M, mesenchyme; VD, ventral diencephalon; PL, posterior lobe; IL, intermediate lobe; AL, anterior lobe. Scale bars: 250 μm (**A** and **B**), 100 μm (**D**). For insets in **A**, original magnification, ×2.7.

The observation of variability in penetrance of the *Dchs2^–/–^* null phenotypes suggested that there might be some functional redundancy compensating for the loss of *Dchs2*. Indeed, DCHS1 and DCHS2 act cooperatively during kidney development, acting with FAT4 as a ligand-receptor pair (24, 26). This led us to explore the possibility that other members of the FAT/DCHS family might additionally contribute to pituitary development. We previously detected strong expression of *Fat4* in the rostral tip of the developing murine pituitary (which develops into the pars tuberalis) and strong expression of *Dchs1* in surrounding mesenchyme (27). To characterize their expression during late pituitary development, we carried out RNAscope mRNA in situ hybridization using specific probes against *Fat4* and *Dchs1* in sagittal sections through WT pituitaries at 18.5 dpc. *Fat4* expression was strong in the developing pars tuberalis, the developing posterior lobe, and the pituitary stalk, and transcripts were also detected in the surrounding mesenchyme and scattered cells of the developing anterior lobe. Low levels of *Dchs1* transcripts were detected throughout these tissues with the highest expression in the pituitary stalk (Figure 4A). To investigate whether *Dchs1* expression increases in *Dchs2^–/–^* mutants, indicative of a compensatory mechanism, we carried out qRT-PCR on whole pituitary lysates comparing *Dchs2^–/–^* and *Dchs2^+/–^* control genotypes. Although not significant with the available samples, there appeared to be an elevation of *Dchs1* mRNA levels in *Dchs2^–/–^* mutants (Supplemental Figure 2B, n** = 4 per genotype).

We hypothesized that loss of FAT4 or DCHS1 could also lead to pituitary defects. Because neither *Fat4^–/–^* nor *Dchs1^–/–^* mutants are viable past the early postnatal period, analysis was limited to embryonic stages and the perinatal period (P0–P2). As in *Dchs2^–/–^* mutants, histological analysis of *Fat4^–/–^* mutants at 13.0 dpc revealed abnormal invaginations in the epithelium of the infundibular recess in 7/10 embryos (arrowheads in Figure 4B). We also observed a severely abnormal invagination of the infundibulum lacking a central lumen, using mRNA in situ hybridization against *Fat3* to mark infundibular tissue (27) (1/10 embryos, Figure 4C). These infundibular anomalies were not observed in *Dchs1^–/–^* embryos (0/5 at 13.0 dpc). The infundibular phenotypes shared between *Fat4^–/–^* and *Dchs2^–/–^* mutants suggest that FAT4 may be acting in concert with DCHS2, as a receptor-ligand pair, during posterior pituitary development.

Upon gross examination at P0, both *Fat4^–/–^* and *Dchs1^–/–^* pituitaries exhibited shortening of the medio-lateral axis of the anterior pituitary compared with WT littermates (Figure 4C), unlike *Dchs2^–/–^* mutants, which did not display this phenotype (*n* = 9, Supplemental Figure 2A). The size of the intermediate and posterior lobes was comparable to WT controls (*n* = 8 for *Fat4^–/–^*, *n* = 10 for *Dchs1^–/–^*). Analysis of proliferation by Ki-67 immunostaining did not reveal differences in the number of cycling cells between genotypes (Supplemental Figure 3, n** = 3 per genotype). Immunofluorescence staining of lineage commitment and differentiation markers of the anterior pituitary (PIT1, SF1, TPIT, GH, TSH, ACTH) identified normal distribution of committed and differentiated cell types in *Fat4^–/–^* pituitaries compared to controls (*n* = 3, Figure 4D). No differences were observed between *Fat4^–/–^* and *Dchs1^–/–^* genotypes (not shown).

## Discussion

Screening 28 patients with EPP and/or PSIS, we have identified 7 variants in *FAT2* and *DCHS2* in 6 patients. Five of these variants were predicted to be damaging by in silico analysis. Indeed, the existence of familial cases with mutations and/or single nucleotide variants in genes involved in the developmental process such as in *HESX1*, *LHX4*, *PROP1*, *OTX2*, *SOX3*, *PROKR2*, and *GPR161* have suggested a Mendelian form of inheritance (6, 7, 29). Recently, Zwaveling-Soonawala et al. also identified *DCHS1* as one of the candidate genes for sporadic PSIS in 2 young patients: a 9-year-old girl who presented with absent stalk and anterior pituitary, EPP, and hormonal deficiencies and a 2.5-year-old boy with small stalk and anterior pituitary, EPP, and hormonal deficiencies (8). Although no functional studies were performed, both these patients had variants in other genes that were also predicted to be damaging, such as in *GLI2*, which has been reported to be involved in holoprosencephaly and abnormal pituitary development (30), and in *BMP4*, which has a crucial role during embryonic pituitary development (31), indicating that variants in one or more genes other than *FAT2* and *DCHS2* may be required for an apparent phenotype.

In our screen, all patients with *FAT2* or *DCHS2* variants had GHD, and 3 of them had combined hormone deficiency. Two of these patients had a more severe phenotype with complete deficiency of the adenohypophysis and a pituitary height smaller than expected for their age and sex.

Considering that in all *Dchs* and *Fat* mouse mutants analyzed, anterior cell type commitment and differentiation occurred normally, the hormonal deficiencies identified in our patients could be due to the lack of trophic signals of the hypothalamic releasing factors rather than defects in the differentiation of the anterior endocrine cells. In the patients, we were not able to identify a specific phenotype-genotype correlation, which indicates that these variants can be confounding factors, or causative where other factors also contribute to the severity of symptoms.

Expression analysis of both mouse and human tissue revealed that in development, FAT/DCHS protocadherins are expressed in the mesenchyme surrounding the pituitary. The infundibulum and posterior pituitary gland receive a rich blood supply from the superior hypophyseal artery, infundibular artery, and inferior hypophyseal artery (32), all of mesenchymal origin (28, 33). Disruption of the pituitary vascular network can result in abnormal tissue morphology and poor function (28, 33). We postulate that mutations in *FAT2* and *DCHS2* may affect the development of the posterior lobe and its connection to the hypothalamus, through affecting the surrounding mesenchymal contribution. By analyzing developing *Dchs2*^–/–^ mouse mutant pituitaries, we have identified a partially penetrant pituitary stalk defect as well as anterior pituitary hypoplasia. Like *Fat2^–/–^* mice, however, these have previously been described as normal, healthy, and viable, possibly due to the redundant roles with other FAT and DCHS family members (26, 34). Consistent with this stalk phenotype, we also identified abnormal infundibular development in *Fat4^–/–^* mutants, previously shown to exhibit poor cerebral neuronal migration (14, 35). As such, the ventral migration of axons of hypothalamic neurons located in the paired supraoptic and paraventricular nuclei, which terminate in the posterior pituitary (4), might also be affected by loss of FAT/DCHS protocadherins, resulting in abnormal evagination of this tissue and PSIS.

Interestingly, analysis of *Fat4^–/–^* as well as *Dchs1^–/–^* single mutants revealed an additional pituitary defect, shortening of the medio-lateral axis of the anterior lobe. As this is consistent between the 2 genotypes, we conclude that FAT4 and DCHS1, expressed in the developing RP, infundibulum, and surrounding mesenchyme (27), are likely acting as a receptor-ligand pair in the morphogenesis of the anterior lobe, and we hypothesize that expression of both proteins in the surrounding mesenchyme is critical for this process (27). FAT4/DCHS1 may be involved in cell movements during development, which is consistent with their known role in planar cell polarity (10, 11, 25), often an important pathway during dynamic rearrangement and migration of cells. In the anterior pituitary they are unlikely to act upstream of YAP for this purpose, as previous work deregulating YAP levels during development did not result in medio-lateral morphogenesis defects (36).

Taken together, our studies have revealed a requirement for the concerted action of FAT/DCHS protocadherins for normal pituitary development and support the pathogenicity of *FAT2* and *DCHS2* variants in patients with ectopic PSIS in addition to other genes reported (8). As PSIS may present with isolated or multiple hormonal deficiencies, neonatal hypoglycemia, micropenis, and/or cryptorchidism with or without growth deficit should prompt early screening with hormonal and imaging investigations for early detection and treatment. Furthermore, genetic screening for identification of a specific mutation in the genes involved in PSIS development is a prerequisite for genetic counseling and appropriate long-term follow-up, which is imperative as there is a strong possibility of development of other hormonal insufficiencies.

## Methods

### WES of patients with EPP.

The data from 28 unrelated patients diagnosed with nonsyndromic EPP were reviewed, and DNA was extracted from peripheral blood mononuclear cells. WES and variant calling were performed for *FAT1*, *FAT2*, *FAT3*, *FAT4*, *DCHS1*, and *DCHS2*. DNA samples from 156 in-house parents of osteosarcoma patients and 8554 samples from the ARIC database were used as controls.

### Analysis of the identified variants.

The variants that were identified in controls were filtered, and analysis was focused on the variants with functional types “high” and “moderate.” Allele frequencies identified in patients with EPP were compared with the allele frequency published on a reference database. The gnomAD data sets span 125,748 exome sequences and 15,708 whole-genome sequences from unrelated individuals and are publicly available online (https://gnomad.broadinstitute.org). The possible functional impact of an amino acid substitution was predicted by 3 different in silico prediction tools: (a) the PolyPhen (Polymorphism Phenotyping) program (http://genetics.bwh.harvard.edu/pph2), which calculates the position-specific independent counts score that represents the probability that a substitution is damaging; values nearer to 1 are more confidently predicted to be deleterious; (b) for missense variants, the SIFT program (http://sift.jcvi.org) calculates normalized probabilities for all possible substitutions from the alignment; positions with normalized probabilities less than 0.05 are predicted to be deleterious; those greater than or equal to 0.05 are predicted to be tolerated; and (c) the CADD (v1.3, https://www.ncbi.nlm.nih.gov/pubmed/30371827), which combines deleteriousness predictions from multiple algorithms into a single phred-like score for all possible single nucleotide variants in the genome (i.e., a CADD score > 10 is predicted to be among the top 10% most deleterious single nucleotide variants in the genome, CADD score > 20 is among the top 1%, CADD score > 30 is among the top 0.1%, etc). For convenience we classified the variants with a CADD score above 20 as “likely deleterious” and below 20 as “likely benign.”

### Animals.

*Dchs2^–/–^*, *Fat4^fl/fl^*, and *Dchs1^–/–^* animals were previously described (24–26). For the generation of *Fat4^–/–^* animals, *Fat4^fl/fl^* mice were crossed with the *Actb^Cre/+^* strain for ubiquitous deletion (37) and offspring were intercrossed. For embryo collection, the morning of vaginal plug was considered 0.5 dpc. Embryos or postnatal pituitaries were dissected, fixed in 10% neutral buffered formalin overnight with agitation, and then dehydrated through an ascending ethanol series. Tissues were processed for paraffin embedding and sectioned to 4 μm for RNAscope mRNA in situ hybridization, or 7 μm for hematoxylin and eosin staining and immunofluorescence.

### Hematoxylin and eosin staining.

Slides were dewaxed in Histo-Clear (National Diagnostics) and rehydrated through a descending ethanol series in distilled H_2_O. Sections were stained with Harris’ hematoxylin and eosin following standard protocols. Slides were dehydrated and dried, then coverslips mounted with VectaMount (Vector Ltd).

### RNAscope mRNA in situ hybridization.

Sections were processed and stained as described previously (27). Briefly, slides were heated to 60°C, dewaxed in xylene, washed in 100% ethanol, and processed following the RNAscope 2.5HD Reagent Kit-RED assay kit (Advanced Cell Diagnostics) with specific probes (*Fat4*, *Dchs1, Fat3*). Following detection, slides were weakly counterstained with hematoxylin and coverslips mounted with VectaMount (Vector Ltd).

### Immunofluorescence.

Slides were deparaffinized in Histo-Clear and rehydrated through a descending ethanol series, followed by antigen retrieval in pH 6.0 citrate buffer in a NXGEN Decloaking Chamber (Menari Diagnostics) at 110°C for 3 minutes. Slides were washed in PBST (PBS with 0.1% Triton X-100), blocked in blocking buffer (0.15% glycine, 2 mg/mL BSA, 0.1% Triton X-100 in PBS) with 10% sheep serum for 1 hour at room temperature, and then incubated overnight in primary antibody in blocking buffer with 1% sheep serum at 4°C. Primary antibodies used were against: DCHS2 (1:500, Atlas HPA064159), FAT2 (1:1000, Santa Cruz Biotechnology sc59985), Ki-67 (1:300, Abcam ab16667), SOX2 (1:300, Abcam ab97959), PIT1 (1:1000, gift from Simon Rhodes, University of North Florida, Jacksonville, Florida, USA), TPIT (1:300, gift from Jacques Drouin, University of Montreal, Montreal, Quebec, Canada), SF1 (1:300, Life Technologies, Thermo Fisher Scientific, N1665), ACTH (1:300, Fitzgerald 10C-CR1096M1), GH (1:1000, NHPP AFP5641801), and TSH (1:1000 NHPP AFP-1274789). The VECTOR Laboratories ImmPRESS kit was used for antibodies against SF1 according to the manufacturer’s instructions. The PerkinElmer TSA kit was used for antibodies against DCHS2 and FAT2, as previously described (36). After primary antibody incubation, slides were washed in PBST, then incubated with appropriate secondary antibodies (biotinylated goat anti-rabbit, 1:300, Abcam ab6720; biotinylated goat anti-mouse, 1:300 Abcam ab6788) and incubated with fluorophore-conjugated streptavidin (1:500, Life Technologies, Thermo Fisher Scientific, S11223) for 1 hour at room temperature. Quenching of endogenous autofluorescence was carried out by treatment with Sudan Black B (MilliporeSigma) following immunofluorescence against TPIT and SF1. Nuclei were counterstained with Hoechst (1:10,000, Life Technologies, Thermo Fisher Scientific, H3570). Coverslips were mounted with VectaMount (VECTOR Laboratories, H1000).

### Imaging.

Wholemount images were taken with an MZ10 F Stereomicroscope (Leica Microsystems), using a DFC3000 G camera (Leica Microsystems). For bright-field images, stained slides were scanned with Nanozoomer-XR Digital slide scanner (Hamamatsu) and images processed using Nanozoomer Digital Pathology View. Immunofluorescence staining was imaged with a TCS SP5 confocal microscope (Leica Microsystems) and images processed using Fiji (38).

### Proliferation analysis.

The number of Ki-67^+^ cells and total nuclei stained with Hoechst were counted manually using Fiji (at least 5000 nuclei counted over 5–7 sections/biological sample) for control and mutant pituitaries; *n* = 3 per genotype. Nuclei along the marginal zone epithelium were recorded separately from those within the anterior pituitary parenchyme.

### qRT-PCR.

Whole pituitaries were dissected from 10-week-old *Dchs2^+/–^* (control) and *Dchs2^–/–^* (mutant) mice and placed in RNA*later*-ice (Thermo Fisher Scientific, AM7030). They were flash-frozen in liquid nitrogen and stored at –80°C. mRNA was extracted using Monarch Total RNA Miniprep kit (New England Biolabs, T2010S) and translated using QuantiTect Reverse Transcription kit (QIAGEN, 205311); then qRT-PCR was performed using QuantiNova SYBR Green RT-PCR kit (QIAGEN, 208152) on a Roche Lightcycler 480. Data were analyzed using ΔΔCT method normalized to housekeeping gene expression; *n* = 4 pituitaries per genotype. Primers used: *Pou1f1* (*Pit1*) forward CACGGCTCAGAATTCAGTCA, reverse TCCAGAGCATCCTTAGCAGC; *Tbx19* (*Tpit*) forward TGTCTCGCCTGCTTAACGTG, reverse GACAGGGAACATCCGTCTGC; *Nr5a1* (*Sf1*) forward AGCTGCAAGGGCTTCTTCAA, reverse CATTCGATCAGCACGCACAG; *Sox2* forward GAGGGCTGGACTGCGAACT, reverse TTTGCACCCCTCCCAATTC; *Dchs1* forward TCCACGTTCATCCACTCAGC, reverse GGGGACTGTTCTCACGAAGG; *Hprt* (housekeeping) forward GTTGGGCTTACCTCACTGCT, reverse TCATCGCTAATCACGACGCT; *Actb* (housekeeping) forward TTCTTTGCAGCTCCTTCGTT, reverse ATGGAGGGGAATACAGCCC.

### Statistics.

Data were analyzed using 2-tailed unpaired Student’s *t* tests using the Holm-Šídák method to correct for multiple analyses, where *P* < 0.05 was taken to be statistically significant.

### Study approval.

All participants, or their legal guardian, provided written and informed consent. The present studies in humans were reviewed and approved by the Irmandade da Santa Casa de Misericórdia de São Paulo review board (project number 34003914.0.0000.5479), located at Marquês de Itu Street, 381, São Paulo, Brazil. Animal husbandry was carried out in compliance with the Animals (Scientific Procedures) Act 1986, relevant Home Office License (P5F0A1579), and KCL Ethical Review approval.

## Author contributions

FH, CAS, and CLA designed research studies; EJL, PX, AS, TSS, FRF, DS, and ALP conducted experiments; EJL, PX, TSS, CK, CAL, AS, JLM, NP, JL, and CLA acquired data; EJL, PX, AS, and FRF analyzed data; TH, PFW, FH, and CAS provided reagents; PX, EJL, and CLA wrote the manuscript; and PFW, FH, JLM, and CAS edited the manuscript.

## Supplementary Material

supplemental data

## Figures and Tables

**Table 1:**
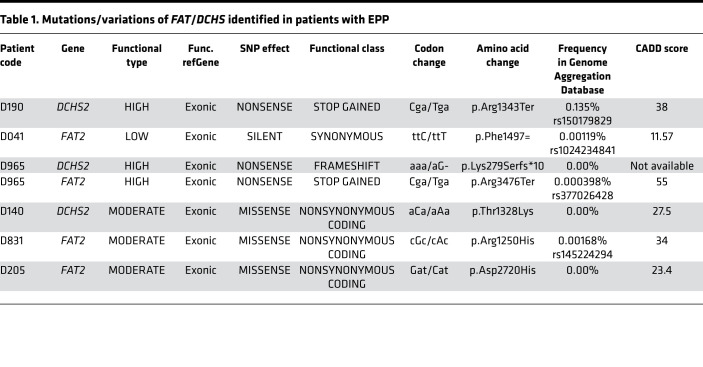
Mutations/variations of *FAT*/*DCHS* identified in patients with EPP

**Table 2:**
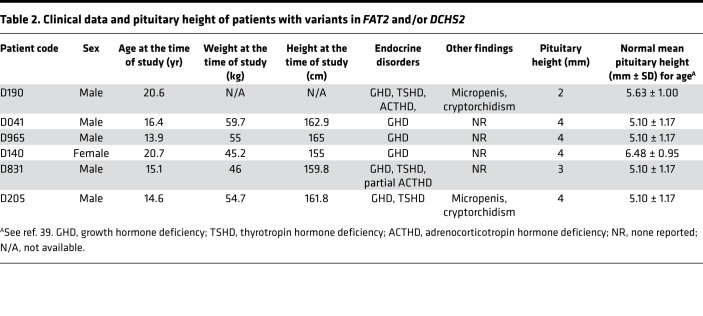
Clinical data and pituitary height of patients with variants in *FAT2* and/or *DCHS2*

## Version 1 10/27/2020

In-Press Preview

## Version 2 12/03/2020

Electronic publication
