# Supplementary material for: Requirement of FAT and DCHS protocadherins during hypothalamic-pituitary development
Source: JCI Insight. 2020 Dec 3;5(23):e134310. doi: 10.1172/jci.insight.134310 (PMC7714405; doi:10.1172/jci.insight.134310)
Supplement: supplemental data [file jciinsight-5-134310-s007.pdf]

Lodge *et al.*

Supplementary Figure 1

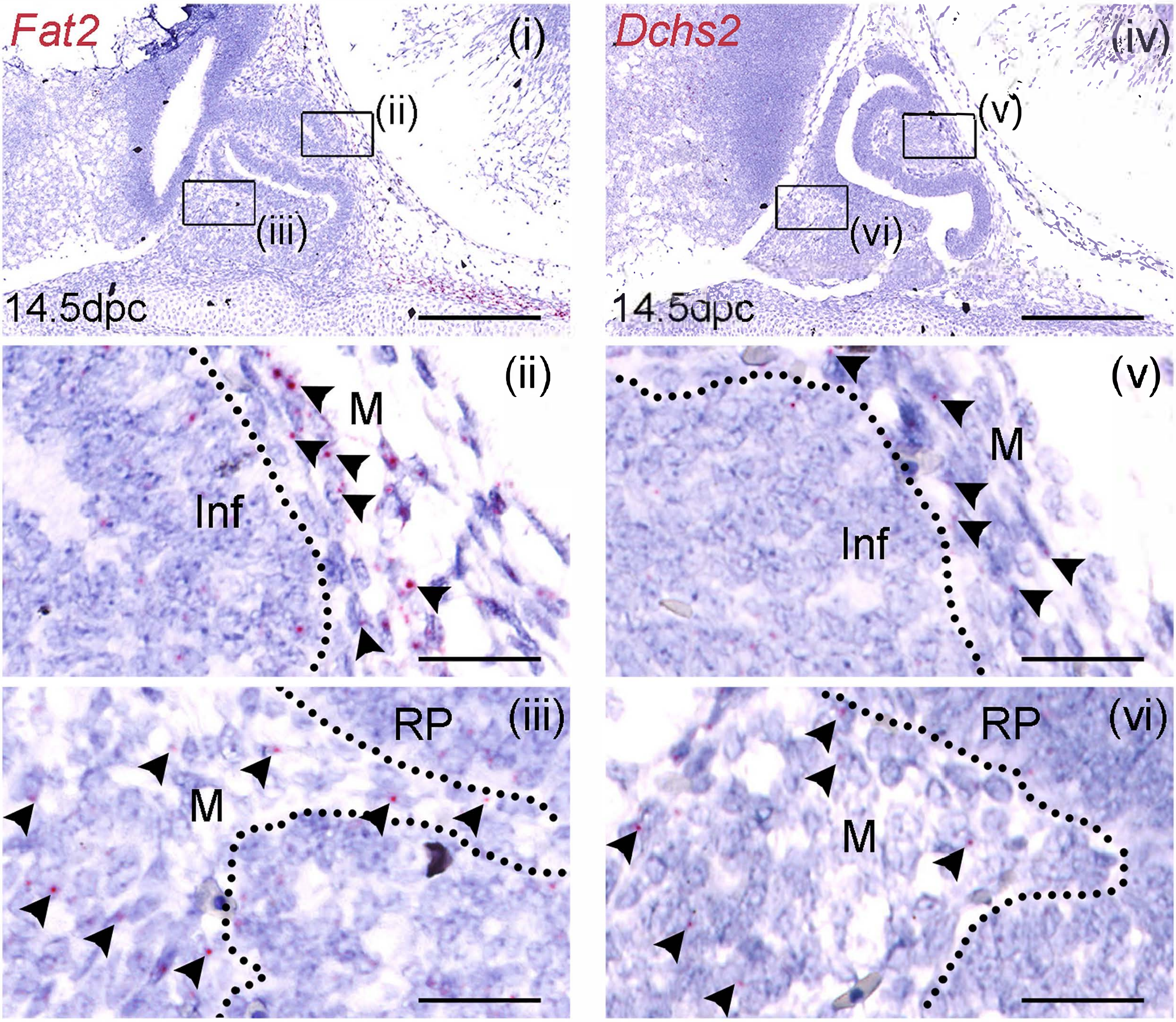

Supplementary Figure 1. Expression of *Fat2* and *Dchs2* in the developing mouse pituitary. RNAscope mRNA *in situ* hyridization using specific probes against *Fat2* and *Dchs2* on sagittal sections of 14.5dpc wild type murine pituitaries (n=3). Red dots (specific staining, arrowheads) are seen in the mesenchyme (M) surrounding the infundibulum (Inf) and developing Rathke's pouch (RP), which is demarcated by dotted lines. Sparse expression is detected within the developing RP. Scale bars 250μm in (i) and (iv), 50μm in (ii), (iii), (v) and (vi).

Supplementary Figure 2

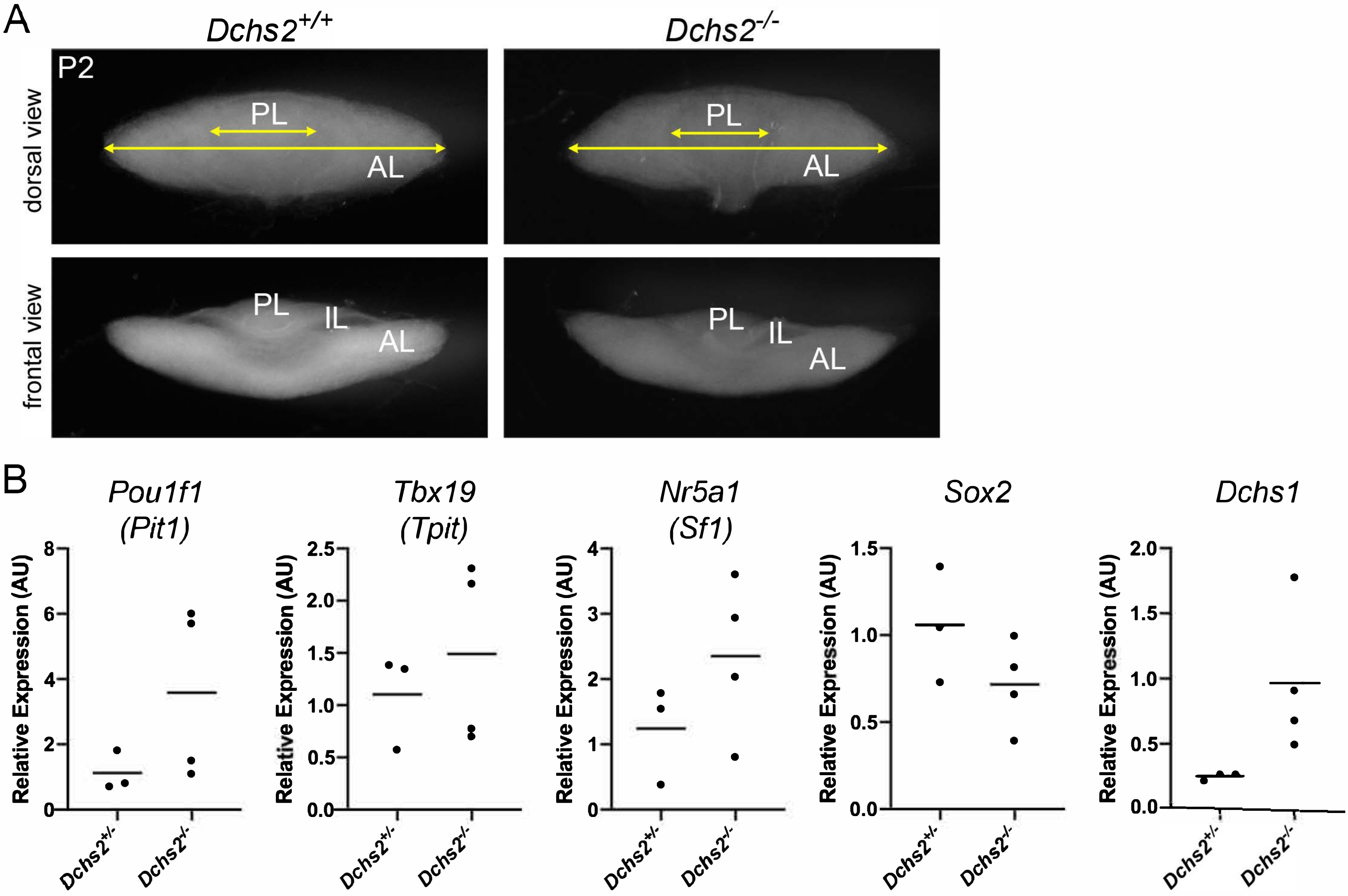

Supplementary Figure 2. Loss of DCHS2 does not impair anterior pituitary cell type commitment or anterior lobe morphology. (A) Wholemount images of dorsal and frontal views (top and bottom panels respectively) of *Dchs2*$^{+/+}$ and *Dchs2*$^{-/-}$ pituitaries at P2, showing comparable morphology (n=9). PL: Posterior Lobe, IL: Intermediate Lobe, AL: Anterior Lobe. **(B)** Relative expression of pituitary differentiation markers *Pou1f1* (*Pit1*), *Tbx19* (*Tpit*), *Nr5a1* (*Sf1*), stem cell marker *Sox2*, and *Dchs1* by quantitative PCR on lysates of wild type control and *Dchs2*$^{-/-}$ pituitaries at 10 weeks (n=3 *Dchs2*$^{+/-}$, n=4 *Dchs2*$^{-/-}$). No statistically significant difference was identified between mutant and control samples for any marker. Student's t-test *Pou1f1* (*Pit1*) *P*=0.0862, *Tbx19* (*Tpit*) *P*=0.163, *Nr5a1* (*Sf1*) *P*=0.340, *Sox2* *P*=0.443, *Dchs1* *P*=0.811.

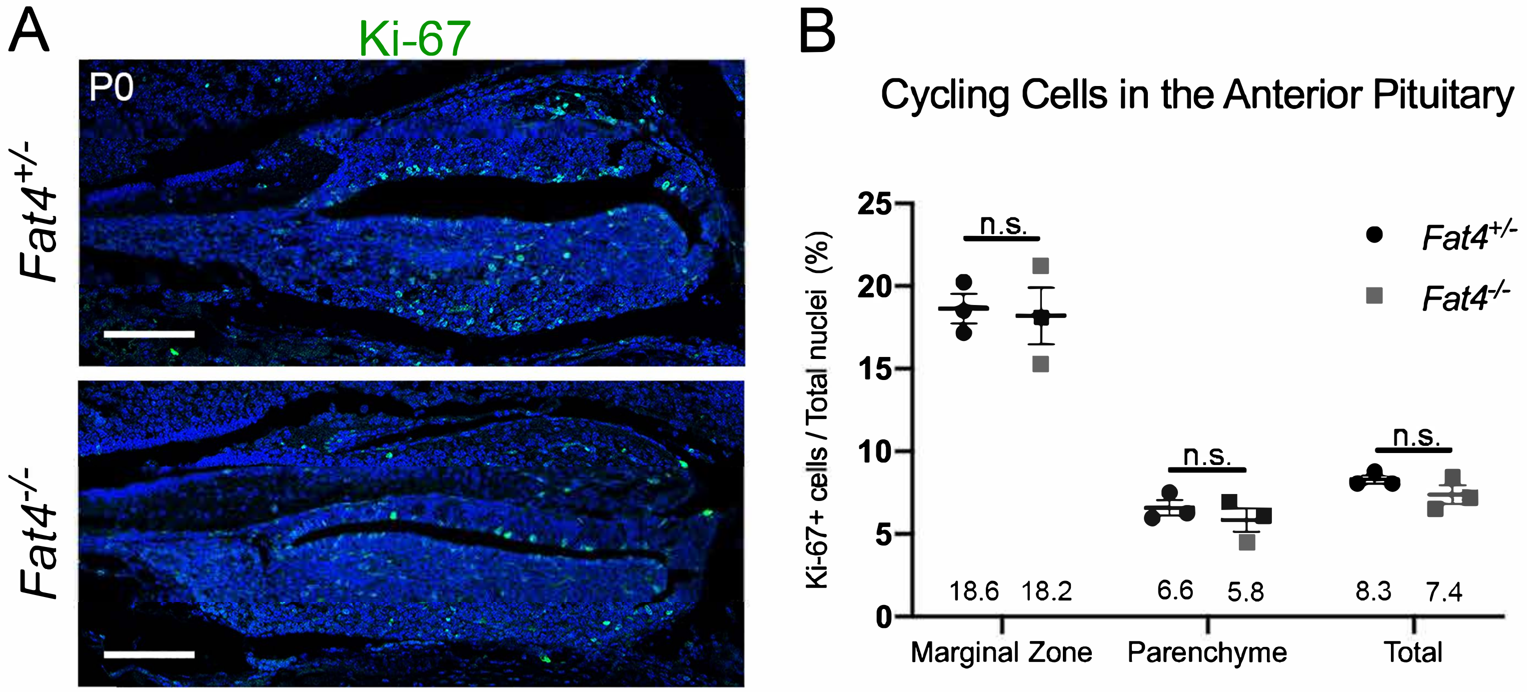

Supplementary Figure 3. *Fat4*$^{-/-}$ pituitaries have normal proliferation at P0.
(A) Immunofluorescence staining using antibodies against Ki-67 to detect cycling cells on sagittal sections of P0 *Fat4*$^{+/-}$ and *Fat4*$^{-/-}$. **(B)** Graph depicting quantification of cycling cells in the anterior pituitary, showing comparable numbers of cycling cells between genotypes, *P*>0.05 unpaired Student's t-test (n=3). Values of Ki-67 positive cells are expressed as a percentage of the total nuclei in the anterior lobe. Average values are indicated. Scale bars 100µm.
